# When work interferes with life

**DOI:** 10.5271/sjweh.4188

**Published:** 2024-10-01

**Authors:** Reiner Rugulies

**Affiliations:** 1 National Research Centre for the Working Environment (NFA), Copenhagen, Denmark.; 2 Department of Public Health, University of Copenhagen, Denmark.

This issue of the Journal contains an article by Gynning et al (1) on the impact of work-life interference on burnout and job discontent in a sample of 1575 physicians in Sweden. The authors measured work-life interference and burnout at baseline in 2021, followed participants for one year, and then measured burnout again. Higher levels of work-life interference in 2021 were associated with an increased risk of high burnout in 2022, after adjustment for sex, occupational rank, family situation, work hours, overtime work, work with COVID-19 patients, and high burnout in 2021. The estimates for the association were substantial, with odds ratios of 3.67 [95% confidence interval (CI) 2.78-4.83] and 1.53 (95% CI 1.05-2.25) in the crude and the adjusted analysis, respectively. Work-life interference in 2021 was also associated with risk of job dissatisfaction and turnover intention in 2022.

Whereas the design of the study was simple and straightforward, the researched phenomena, both the exposure − work-life interference − and the outcome − burnout − are anything but simple and straightforward.

## Work-life interference

Work-life interference and related concepts, such as work-life balance or work-family conflict, have been viewed and understood differently, depending on historical and other contexts. In the beginning of the Industrial Revolution, the demand for a healthy balance between work and life might have been best expressed in the slogan “eight hours labor, eight hours recreation, eight hours rest”, allegedly coined by the British textile manufacturer and social reformer Robert Owen (1771–1858). Although the link between number of working hours and work-life balance persisted in the 21^st^ century − as illustrated in a review by Albertsen et al (2) on working hours and work-life balance published in this journal in 2008 − new aspects emerged, such as the role of gender and gender disparities. Albertsen et al reported in their review that long working hours were strongly associated with a risk of lower work-life balance among women, whereas among men the association was much less clear. Today, there is a substantial literature studying and critically discussing concepts such as work-life interference and work-life balance from a feminist and post-feminist perspective (3, 4).

In recent years, a generational perspective has been added to the research on work-life interference. It has been argued that workers from Generation Y (Gen Y or Millennials), comprising those born between the early 1980s and the mid-to-late 1990s, and Gen Z, those born between the mid-to-late 1990s and early 2010s, are more sensitive about the negative effects of work on life and focused on protecting themselves from work that interferes with life than previous generations (such as Gen X or Baby Boomers) (5, 6). Other researchers, though, have strongly criticized the generational perspective and argue there are little-to-no generational effects and that the apparent differences between the generations are actually due to age and period effects (7).

An important period effect might be that in this day and age, the distinction between work and life is becoming less clear compared to earlier days of industrialized societies. Whether this is good or not for workers’ physical and mental health is not easy to answer. For many workers, and in particular but not limited to highly educated professionals (such as us, academic researchers), work is a major part of life and a key contributor to identity and self-esteem. The domains of work and life are often so much entangled that terms such as “interference” or “balance” may not be adequate to describe the relation between the two. The distinction between work and life may be further blurred because of technological advancements that allow a growing proportion of workers to work from home.

## Burnout

The term burnout emerged in the 1970s from research on physical and mental exhaustion among volunteer workers in the USA (8). It gained popularity in particular due to the work by Christina Maslach, professor of psychology at the University of California at Berkeley, and the development of the Maslach Burnout Inventory (9). Maslach conceptualized burnout with three dimensions: emotional exhaustion, depersonalization (also termed cynicism), and reduced personal accomplishment (also termed reduced professional efficacy) (9). While the Maslach burnout inventory is by far the most widely-used instrument to assess burnout, it has also been criticized and controversially discussed, in particular with regard to the dimensions of depersonalization and reduced personal accomplishments (10, 11).

Several other instruments, definitions, and conceptualizations of burnout have been proposed over the decades (12). A few years ago, the Network on the Coordination and Harmonisation of European Occupational Cohorts (OMEGA-NET) set up a panel of 50 researchers and healthcare professionals, with the aim of developing a consensual definition of occupational burnout. The results were published in the Journal in 2021 (12). The consensus definition was: “In a worker, occupational burnout or occupational physical AND emotional exhaustion state is an exhaustion due to prolonged exposure to work-related problems” (12). This brief consensus definition was the result of the panel's impressive work that included reviews of the literature, critical discussions of the findings, and thoughtful deliberations. However, already in the next issue of the Journal, the consensus definition was challenged. In an editorial, Wilmar Schaufeli concluded that several questions regarding burnout still remain unsolved (13). One question is whether “exhaustion” is sufficient to define burnout, as the panel argued, or, as Schaufeli argued, that the burnout concept requires the inclusion of both the inability to spend effort at work (exhaustion) and the unwillingness to spend this effort (what Schaufeli termed “mental distancing”). Gynning et al (1) seem to have followed Schaufel's reasoning and used in their study the Burnout Assessment Tool 12 (BAT-12) (14), a measurement that includes items both on exhaustion and mental distancing (see the supplementary material of their article for the wording of the items).

## Prevalence of burnout among physicians

When one searches the Web of Science for the topics “work-life interference" OR "work-life balance”, remarkably, the top-five cited articles that show up are all about burnout among physicians (15–19). It is further notable that each of these five articles had more than 1000 citations even though they were relatively recently published: in 2018 (two articles), 2016, 2015, and 2012, respectively. Thus, it seems fair to conclude that the study by Gynning et al addresses an area of great contemporary interest.

Is prevalence of burnout particularly high among physicians compared to other occupational groups? The seminal article by Shanafelt et al (15) reported that 45.8% of the physicians showed at least one burnout symptom on the Maslach Burnout Inventory. For comparison with a US population control sample, Shanafelt and colleagues used a 2-item burnout measure that yielded a burnout prevalence of 37.9% among physicians and 27.8% among controls.

Recently, Møller et al (20, 21) presented results from a burnout study that invited all 104 active vascular surgeons (including vascular surgeons in training) employed at a department of vascular surgery in a Danish hospital. Of those 104 surgeons, 85 completed the survey, yielding an astonishing response rate of 82%. Burnout was measured with the Copenhagen Burnout Inventory, which assesses exhaustion (but not mental distancing) on three separate scales: personal burnout (exhaustion without attribution to a cause), work-related burnout (exhaustion attributed to work), and client/patient-related burnout (exhaustion attributed to work with clients or patients (10). The prevalence of moderate or severe burnout was 28%, 16% and 4% for personal, work-related, and client/patient-related burnout, respectively (20).

Compared to the burnout prevalence reported by Shanafelt et al (15) and Møller et al (20), the burnout prevalence reported by Gynning et al is rather low (4.5% and 5.8% in 2021 and 2022, respectively). There might be many explanations for these huge differences in burnout prevalence across the three studies, such as different response rates and bias due to selective non-response or differences in working conditions between the USA, Denmark and Sweden. The most likely explanation, though, is probably the use of different instruments to measure burnout and thereby different definitions of burnout used in the three studies. Thus, for comparative studies, across countries, industries, or job group, a harmonization of burnout measurements is needed.

## Mechanisms

As delineated above, work hours and overtime work are seen as crucial components of work-life interference in the literature (2). It is therefore remarkable that Gynning et al reported that the association between work-life interference and burnout remained, even after adjusting for work hours and overtime work. So, what is it in work that is interfering with life if not work hours and overtime? The five items to measure work-life interference in the study by Gynning et al are rather general: (i) feeling tired when coming home from work; (ii) private life is not as desired because of work; (iii) overlook personal problems because of demands at work; (iv) personal life suffers because of work; (v) change and adapt personal life to demands at work (see the Supplementary Material of the article for the exact wording of the items). The demands at work that are mentioned in the items could be quantitative demands, including work intensity, but also emotional demands, which tend to be high among physicians (22). It is also possible, though, that the respondents viewed “demands” more broadly and subsumed under the term interpersonal conflicts at work or poor work organization.

In an invited commentary to Møller et al's (21) study on burnout among vascular surgeons, Jonathan Meizoso, a US-American physician, discussed possible explanations for the high burnout prevalence in the study (23). He first expressed his puzzlement that so many Danish vascular surgeons reported burnout, in particular since the working conditions of the Danish physicians seem to be so much better compared to their US-American counterparts (37-hour regular work week, paid overtime work, paid sick leave, paid maternity and paternity leave, six weeks of paid vacation). But then he pointed to adverse working conditions listed in the article that sounded familiar to him as a physician practicing in the USA, such as “increasing bureaucracy, rising requirements of documentation, and an unfavorable electronic health record system” (23). On a theoretical level, these factors seem to be related to the concepts of “unnecessary and unreasonable tasks” in the “Stress as Offense to Self” framework (24). Future studies on work-life interference and burnout might want to examine these and other work organizational factors as potential determinants of work-life interference. These studies could also investigate the extent to which a possible effect of work organizational factors on the risk of burnout is due to work-life interference and the extent to which such an effect occurs via other mechanisms.
